# Complete Genome Sequences of Five Bacteriophages That Infect *Enterobacteriales* Hosts

**DOI:** 10.1128/mra.01223-21

**Published:** 2022-03-28

**Authors:** Seth Evans, Hunter Cobbley, Kye Davis, Tyler Divis, Nathaniel Eberhard, Samuel Flor, Evan B. Harris, J. Ben Gordon, Matthew Hyer, Weston Larson, Aurora R. Suorsa, Ruchira Sharma, Carson Sork, Daniel W. Thompson, Lauren Wells, Sherwood R. Casjens, Julianne H. Grose

**Affiliations:** a Department of Microbiology and Molecular Biology, Brigham Young University, Provo, Utah, USA; b School of Biological Sciences, University of Utah, Salt Lake City, Utah, USA; c Division of Microbiology and Immunology, Department of Pathology, University of Utah School of Medicine, University of Utah, Salt Lake City, Utah, USA; Loyola University Chicago

## Abstract

Full genome sequences of five bacteriophages that were isolated from raw sewage samples and infect *Enterobacteriales* hosts are presented. Brookers is a P22-like Proteus phage, OddieOddie is a 9g-like Escherichia coli phage, Diencephelon is a Kp3-like Klebsiella phage, and Rgz1 and Lilpapawes are classic T4-like and T7-like virulent Proteus phages, respectively.

## ANNOUNCEMENT

Many *Enterobacteriales* species harbor both harmless and disease-causing strains, making understanding their evolutionary differences of key importance (1). Phages are a major force in bacterial evolution due to their sheer abundance, their ability to transfer genetic elements, their ability to persist as prophages within a host, and their ability to lyse and kill their hosts (1–4). Here, the full genome sequences of five phages that infect *Enterobacteriale*s are reported.

All five phages (Table 1) were isolated from raw sewage samples collected from water treatment plants in the western United States. Raw sewage (0.5 mL) was incubated with a bacterial culture (0.5 mL) and LB medium (4 mL) at 37°C for 48 to 72 h to enrich for phages; bacteria were then pelleted via centrifugation, followed by plating in LB top agar. Single plaques that arose were then picked and replated with bacteria in top agar. This single plaque isolation was repeated at least three times before preparation of liquid lysates (0.5 mL bacterial overnight culture in 4 mL LB medium at 37°C for 48 to 72 h before pelleting of bacteria by centrifugation). DNA was purified from lysates (>10^8^ PFU/mL) using the phage DNA isolation kit (Norgen Biotek, Canada), prepared for Illumina HiSeq 2500 paired-end 250-bp sequencing using the Illumina TruSeq DNA Nano kit, and assembled *de novo* using Geneious v.R11 (5) except for phage Diencephalon, which was prepared for Illumina paired-end 150-bp iSeq sequencing using the NEBNext Ultra II DNA kit and assembled using Geneious v.8.0.5. Software was used with default settings. All five genomes circularized upon assembly, indicating complete genomes and a possible headful DNA packaging strategy.

**TABLE 1:** Sequencing summary and basic properties of five *Enterobacteriales* phages

Phage name	Sewage sampling location coordinates	GenBank accession no.	SRA accession no.	Total no. of reads	Fold coverage (range [mean])	Length (bp)	GC content (%)	Taxonomy^*a*^
vB_PmiS_Brookers	40.1652°N, 111.610°W	OL539469	SRR17231387	2,389	13–65 (36.2)	40,152	40.7	P22-like
vB_EcoS_OddieOddie	39.7392°N, 104.9903°W	OL539454	SRR17231351	415,000	972–8,268 (1,726.4)	59,985	44.5	9g-like
vB_KaeS_Diencephalon	40.0444°N, 111.7322°W	OL539440	SRR17231361	45,277	75–227 (144.6)	47,263	56.2	KP3-like
vB_MmoM_Rgz1	40.2338°N, 111.6585°W	OK499989	SRR11628752	195,206	113–424 (290.4)	165,808	34.7	T4-like
vB_MmoP_Lilpapawes	40.0444°N, 111.7322°W	OK499982	SRS6381066	714,839	3,197–13,659 (4,579.3)	39,168	47.1	T7-like

a Taxonomy assigned to previously assigned *Enterobacteriales* phage clusters, as described previously (6).

All five phages display similarity in genome size and sequence to previously defined *Enterobacteriales* phage clusters (6) as determined by BLASTN (7). The genome of one of these phages, Brookers, was isolated using Proteus mirabilis Hauser ATCC 7002 and displays high levels of sequence similarity and gene synteny to the well-known Salmonella temperate phage P22 (GenBank accession number BK000583) (∼73% identity over >18% of the genome). In addition, very similar sequences (>94% identity over >50% of the Brooker’s genome) are found in numerous Proteus genomes, indicating that very similar prophages reside in these strains. We conclude that Brookers is a temperate phage.

Two of these lytic phages, Rgz1 and Lilpapawes, were isolated using Morganella morganii subsp. *morganii* Fulton ATCC 25829 as the host. Rgz1 is a T4-like phage whose closest relatives are *Providencia* phages PSTRCR 127 (GenBank accession number MW358927) and 121 (GenBank accession number MP358300) and *Morganella* phage MP1 (GenBank accession number KX078569) (8) (>80% identity over >77% of the genome). The smaller Lilpapawes is a T7-like *Morganella* phage whose closest relative is *Morganella* phage MP2 (GenBank accession number KX078568) (8) (>95% identity over 86% of the genome).

The remaining two lytic phages, OddieOddie and Diencephelon, were isolated using Escherichia coli BW 25113 (9) and Klebsiella pneumoniae subsp. *pneumoniae* (Schroeter) Trevisan ATCC 10031, respectively. OddieOddie has strong similarity to phages in the 9g-like cluster of *Enterobacteriales* phages (6); in particular, its genome is 94% identical to that of E. coli phage Seurat (GenBank accession number KM236243) (10) in the ICTV genus *Seuratvirus*. The Diencephelon genome has 91% identity to that of Enterobacter phage ATCEA22 (GenBank accession number MW419910). This places Diencephelon in a previously undescribed Kp3-like *Enterobacteriales* phage cluster (J. H. Grose and S. R. Casjens, unpublished data) that contains phages that infect Klebsiella and Enterobacter.

### Data availability.

The accession numbers for all five *Enterobacteriales* phages are found in Table 1.
